# Breast cancer and neurotransmitters: emerging insights on mechanisms and therapeutic directions

**DOI:** 10.1038/s41388-022-02584-4

**Published:** 2023-01-18

**Authors:** Priya Jayachandran, Francesca Battaglin, Carly Strelez, Annika Lenz, Sandra Algaze, Shivani Soni, Jae Ho Lo, Yan Yang, Joshua Millstein, Wu Zhang, Jean C. Shih, Janice Lu, Shannon M. Mumenthaler, Darcy Spicer, Josh Neman, Evanthia T. Roussos Torres, Heinz-Josef Lenz

**Affiliations:** 1 Division of Oncology, Norris Comprehensive Cancer Center, Keck School of Medicine, University of Southern California, Los Angeles, CA US; 2 Lawrence J. Ellison Institute for Transformative Medicine, Los Angeles, CA US; 3 Department of Population and Public Health Sciences, Keck School of Medicine, University of Southern California, Los Angeles, CA US; 4 Alfred E. Mann School of Pharmacy and Pharmaceutical Sciences, University of Southern California, Los Angeles, CA US; 5 Department of Biomedical Engineering, Viterbi School of Engineering, University of Southern California, Los Angeles, CA US; 6 Department of Neurosurgery, Norris Comprehensive Cancer Center, Keck School of Medicine, University of Southern California, Los Angeles, CA US

**Keywords:** Prognostic markers, Breast cancer, Mechanisms of disease

## Abstract

Exploring the relationship between various neurotransmitters and breast cancer cell growth has revealed their likely centrality to improving breast cancer treatment. Neurotransmitters play a key role in breast cancer biology through their effects on the cell cycle, epithelial mesenchymal transition, angiogenesis, inflammation, the tumor microenvironment and other pathways. Neurotransmitters and their receptors are vital to the initiation, progression and drug resistance of cancer and progress in our biological understanding may point the way to lower-cost and lower-risk antitumor therapeutic strategies. This review discusses multiple neurotransmitters in the context of breast cancer. It also discusses risk factors, repurposing of pharmaceuticals impacting neurotransmitter pathways, and the opportunity for better integrated models that encompass exercise, the intestinal microbiome, and other non-pharmacologic considerations. Neurotransmitters’ role in breast cancer should no longer be ignored; it may appear to complicate the molecular picture but the ubiquity of neurotransmitters and their wide-ranging impacts provide an organizing framework upon which further understanding and progress against breast cancer can be based.

## Introduction

**Fig. 1:**
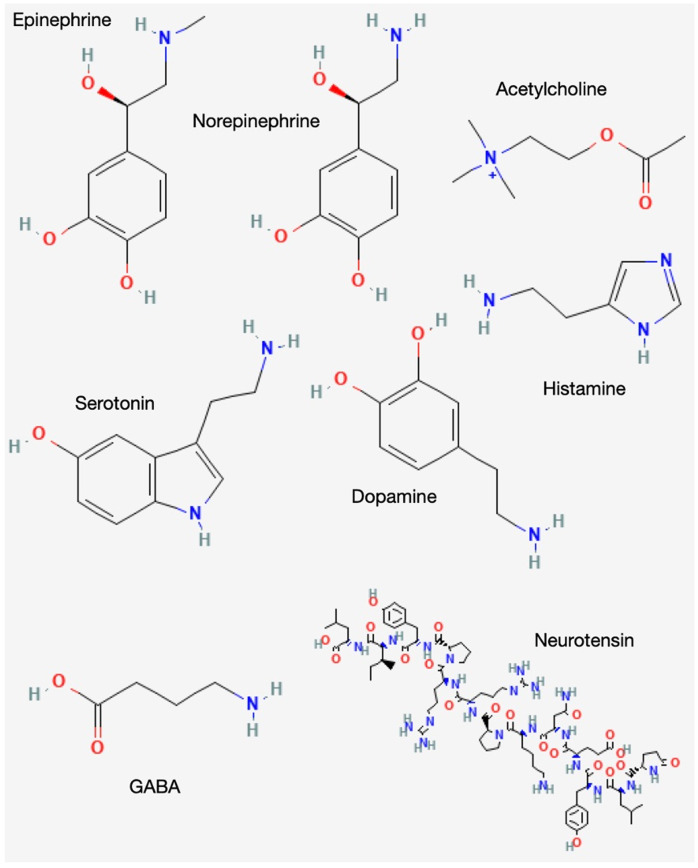
This review discusses a number of neurotransmitters. (Images from PubChem).

Breast cancer is the most common cancer among women. Despite research progress, it remains the leading cause of cancer death among women [1] and much about its molecular biology is still not understood. Neurotransmitters are nerve-secreted substances that modulate neuronal functions by binding to their respective receptors. They play regulatory roles in the physiological functions of tissues and organs and disruptions are associated with various pathologic states (Fig. 1).

**Fig. 2:**
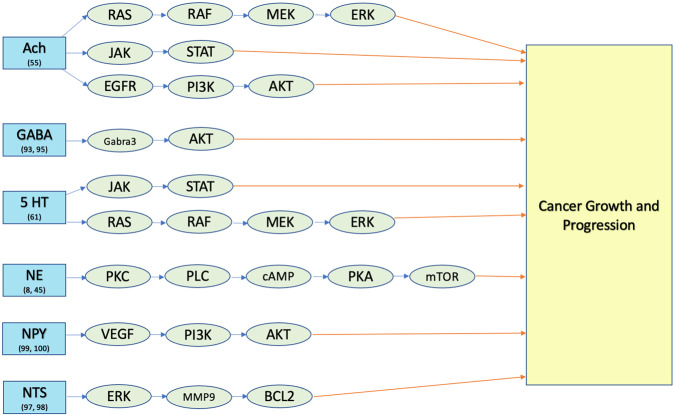
Neurotransmitter signaling pathways in breast cancer metastasis [8, 45, 55, 61, 93, 95, 97–100, 119]. Neurotransmitters such as acetylcholine (Ach), GABA, serotonin (5 HT), norepinephrine (NE), neuropeptide Y (NPY), and neurotensin (NTS) affect cancer progression through multiple signaling pathways.

It has been established in recent decades that neurotransmitters are involved in signaling pathways which influence the course of various malignancies, including breast cancer. Emerging data suggest that cancer cells take advantage of neurotransmitter-initiated signaling pathways, including VEGF, p53, AKT, and MAPK, to activate uncontrolled proliferation and metastasis (Fig. 2). In addition, neurotransmitters can affect immune cells and endothelial cells in the tumor microenvironment via monoamine oxidase modulation of tumor-associated macrophages to promote tumor growth (Table 1).

**Table 1:** Several neurotransmitters and receptors and their effects on target cells and the tumor microenvironment [120].

	Target cells	Increased/Activated	Decreased/Suppressed
Norepinephrine and epinephrine (via ß-adrenergic receptors) (8–11)	Cancer cells	Proliferation, migration, invasion, anti-apoptosis	
	Immune cells	Proinflammatory cytokines	NK cell cytotoxicity, dendritic cell function, lymphocyte response
	Endothelial cells	Angiogenesis	
Acetylcholine (via nicotinic and muscarinic receptors) (53–56)	Cancer cells	Proliferation, migration, invasion, anti-apoptosis, stemness, epithelial to mesenchymal transition	
	Immune cells	Macrophage recruitment, anti-inflammatory response	
	Endothelial cells	Angiogenesis	
Serotonin (via 5-HT receptors) (59–63)	Cancer cells	Proliferation, migration, invasion, autophagy	
	Immune cells	Platelet activation, M2 macrophage polarization	
	Endothelial cells	Angiogenesis	
Dopamine (via dopamine receptors) (82–86)	Cancer cells	Proliferation, migration, invasion (may be increased or decreased)	
	Immune cells		MDSC activation, M2 macrophage polarization, Treg cell activity
	Endothelial cells		Endothelial progenitor cell mobilization, angiogenesis
GABA (via GABA receptors) (94,95)	Cancer cells	Proliferation, migration, invasion (via GABAaR)	Proliferation, migration, invasion (via GABAbR)
	Immune cells	Macrophage recruitment, T cell dependent cytotoxicity	
	Endothelial cells	Unknown	

An appreciation of the role played by neurotransmitters is critical given both the numerous ways that neurotransmitters influence cancer and the number of factors that greatly influence neurotransmitter signaling, ranging from common medications (such as beta blockers and antidepressants) to stress (adrenergic activation) [2] (Table 2). Breast cancer tumors are commonly innervated and greater infiltration of nerve fibers into the tumor microenvironment may predict a worse outcome [3, 4]. The significance of neurotransmitters to breast cancer growth could point the way to lower-cost and lower-risk interventions.

**Table 2:** The explanations for a number of longstanding observations regarding lifestyle likely involve neurotransmitters to some degree.

**Topic**	**References**
Exercise	[107, 108]
Stress	[4, 7, 108–111]
Meditation	[111–113]
Gut microbiota	[114, 115]
Smoking	[53–56]

An exhaustive detailing of the entire landscape of neurotransmitter interactions with cancer is beyond the scope of a single paper. This review emphasizes the following critical topics, selected as likely closest to affecting standards of breast cancer care and representative of the myriad range of neurotransmitter impacts on breast cancer: epinephrine, norepinephrine, and adrenergic activation; acetylcholine; serotonin; dopamine; histamines; gamma-aminobutyric acid (GABA); and neurotensin; and drugs, such as beta blockers and monoamine oxidase inhibitors, impacting pathways mediated by these neurotransmitters (Table 3). Key questions remain open in most of these areas, and further work on these topics holds the potential of great impact to improve our understanding of breast cancer and our therapeutic arsenal against it.

**Table 3:** A large range of medications with other primary indications potentially affect breast cancer positively or negatively via neurotransmitter mediation.

Medication category	Example	References
Beta blockers	Propranolol	[13, 15–38]
Opiate antagonists	Naltrexone	[38, 39]
α2-adrenergic receptor agonists	Dexmedetomidine	[44–49]
α2-adrenergic receptor antagonists	Tramadol	[50, 51, 63]
Muscarinic agonists	Bethanechol	[55, 56]
Tricyclic medications	Amitryptiline	[66, 67]
SSRIs	Fluoxetine	[68–77]
Dopamine receptor agonists	Cabergoline	[82–86]
GABA_A_ receptor agonists	Propofol	[93–95]
MAOIs	Phenelzine	[103–106]

The wide-ranging impacts of neurotransmitters also provide consistent support for seemingly disparate areas of cancer research, ranging from the microbiome to fasting to exercise.

### Adrenergic activation

Epinephrine (adrenaline) and norepinephrine (noradrenaline) are endogenous catecholamines or monoamine neurotransmitters. Release of these hormones mediates the fight-or-flight response as they bind to adrenergic receptors. Breast cancer tissue has been found to overexpress β-adrenergic receptors [5].

Stress induced neuroendocrine activation or pharmacologic activation of adrenergic receptors has been found in mouse models to result in substantial increases to proliferation and distant metastasis [6]. This phenomenon of adrenergic receptors activated by stress neurotransmitters worsening cancer state is not fully understood, but studies are proceeding rapidly [7]. It has also been shown in mice that local mammary tumor sympathetic innervation responds to stress with increased norepinephrine turnover [8] and this is of greater significance to tumor growth than circulating norepinephrine [9]. There are several proposed mechanisms underlying the pro-tumor roles of epinephrine and norepinephrine. One is that activation of β2-adrenoceptor (AR) promotes tumor growth and angiogenesis through increased expression of vascular endothelial growth factor (VEGF), metalloproteases 2 (MMP2), and MMP9. This further potentiates the angiogenic and metastatic evolution of breast cancer [8]. ß_2_AR signaling may also stimulate tumor growth by promoting DNA damage and p53-associated apoptosis suppression [10].

Chances of recurrence appear to be increased by stress. Perego et al. proposed, and supported with murine evidence for lung and ovarian cancer, a mechanism for the awakening of dormant cancer cells [11]. They proposed that pro-inflammatory S100A8/A9 complexes are released by polymorphonuclear neutrophils via β2-adrenergic receptors, in response to stress hormones, and the complexes cause oxidized lipids to accumulate. The release of S100A8/A9 proteins and modified lipids upregulates a fibroblast growth factor receptor pathway, activating dormant cancer cells.

In metastatic breast cancer, the skeleton is a frequent site of spread. Sympathetic nerves densely network across bones. It has been shown that activation of β2-adrenergic receptors, as in sympathetic nerve activation, in osteoblasts leads to increased bone vascular density and a more favorable bone microenvironment for breast cancer cells [12–14].

### Beta blockade

If adrenergic activation stimulates breast cancer progression, then a natural question is whether inhibiting this activation can provide clinical anticancer benefits. This question has been aggressively pursued, particularly given the ready availability of beta blocker drugs [15–18].

Beta blockers are primarily used for cardiac purposes—to treat abnormal heart rhythms and as a second- or later-line treatment for hypertension. Beta blockers are competitive antagonists that block receptor sites for epinephrine and norepinephrine on adrenergic beta receptors. Several of the preclinical studies examining the impact of adrenergic activation on breast cancer growth and migration also tested the impact of beta blockers and found them to have an inhibitory effect [18].

On the clinical side, results have charted a more winding path. A 2017 meta-analysis of six studies, with a cumulative breast cancer patient population over 18,000, found no benefit of beta blockers on overall deaths, cancer-specific deaths, or recurrence [19]. However, this study did not draw conclusions on potential benefits for specific subtypes of breast cancer. Other meta-analyses published over a range of cancers had mixed findings [20–22]. A more recent meta-analysis of 17 studies found no significant association between beta blocker use and breast cancer recurrence [23]. Further, an analysis by Modi et al. found existing beta blocker use at the time of anti-HER2 therapy to actually be associated with worse overall survival among patients with advanced HER2-positive breast cancer [24].

Other studies have reported more promising conclusions, particularly in regards to metastasis and recurrence; this evidence is stronger, though not definitive, for certain cancer subtypes [25]. An examination of 800 women with triple negative breast cancer who took beta blockers found significantly reduced risk of recurrence, metastasis, and death [26]. An additional retrospective breast cancer population study found that beta blockers improved relapse-free survival, though not overall survival, after correcting for differences in cancer severity, hypertension, and other factors [27]. Powe et al., Parada-Huerta et al., and Choy et al. all found beta blockers to be correlated with reduced breast cancer metastasis [28–30]. Spera et al.’s retrospective analysis found an improvement in progression-free survival, particularly for triple negative phenotype and for patients who were not taking a beta blocker prior to cancer treatment [31].

Furthermore, a meta-analysis of studies published between 2010 and 2013 found that women with breast cancer who took beta blockers had a significantly decreased risk of breast cancer death than women with breast cancer who did not take beta blockers [20]. A recent meta-analysis of nearly 15,000 New Zealand breast cancer patients saw a short term—over 3 months—increased risk of death among patients who took beta blockers post-diagnosis, and a protective effect with long-term use [32].

Propranolol is a beta blocker that was approved for use in the US in 1964. It is still in widespread use and it nonselectively blocks β-adrenergic receptors. In a triple-blind placebo-controlled trial reported in 2020, 30 patients with early-stage breast cancer were given propranolol for 7 days prior to resection and 30 were given placebo [33]. Those given propranolol showed reduced intratumoral mesenchymal polarization and increased immune cell infiltration.

Montoya et al.’s study based on a single stage III breast cancer patient found that neoadjuvant propranolol reduces pro-proliferative Ki-67 and pro-survival Bcl-2 markers, and significantly increases p53 expression and induces apoptosis [34]. Montoya et al. had earlier reported that non-selective beta blockers significantly reduced tumor proliferation in early stage breast cancer, based on a retrospective analysis [35]. They also found that a three-week course of propranolol in one patient with early-stage breast cancer was associated with a reduction in Ki67 positive tumor cells, and selective beta blockers were not as effective.

In a randomized placebo-controlled biomarker trial, perioperative inhibition of COX-2 and β-adrenergic signaling was found to inhibit multiple cellular and molecular pathways related to metastasis and disease recurrence in early-stage breast cancer [36]. Combination with neoadjuvant chemotherapy is also being investigated: A phase II trial tested combining propranolol with neoadjuvant taxane/anthracycline-based chemotherapy demonstrated feasibility in the hopes of regulating angiogenesis and reducing distant metastases [37].

The combination of propranolol with non-chemotherapy medications is also showing promise. For example, Murugan et al. investigated the combination of propranolol with naltrexone (an opiate antagonist used to counteract drug and alcohol dependence) and found it to substantially inhibit tumor growth and improve survival in rat xenografts. The effects were attributed to decreasing tumor cell proliferation, inducing cellular apoptosis, and preventing the epithelial–mesenchymal transition in the tumor [38]. In follow-up work, the glycogen synthase kinase 3 pathway was identified as possibly involved in cross talk between β-adrenergic receptors and mu-opioid receptors, with the conclusion that targeting the receptors or glycogen synthase kinase 3 system could prove fruitful in treating triple negative breast cancer [39]. β1-blockers are also starting to gain interest. Nebivolol, which blocks β1-adrenergic signaling, has been found to halt colon and breast tumor growth in xenograft mice [40].

Combating drug resistance is also an area of promise. β2-andrenergic receptors form a positive feedback loop with Her2 in breast cancer cells [41]. Liu et al. found that β2-adrenergic receptors are predictive marker for response to trastuzumab-based therapy in breast cancer, and that propranolol improves the effectiveness of trastuzumab [42]. They also found that it can re-sensitize resistant cells to trastuzumab and, based on a study of medical records, found that treatment with both trastuzumab and beta blockers significantly improved progression-free survival and overall survival in the patients with Her2-positive metastatic breast cancer. Re-sensitization to existing therapies is a much sought after goal in oncology [43].

### α2-Adrenergic receptor agonists

While the interplay with β-adrenergic receptors has received more attention, other adrenergic receptors also appear to be relevant. Szpunar et al. showed that, in mice, treatment with a highly selective α2-adrenergic receptor agonist, dexmedetomidine, increased tumor growth and metastasis [44]. Dexmedetomidine is a common surgical anesthetic, including for breast cancer surgery. Based on in vitro and xenotransplant in vivo assays, Xia et al. reported that dexmedetomidine could increase the proliferation, migration, and invasion of breast cancer cells via activation of α2B-adrenoceptor / ERK signaling [45]. Subsequent studies have corroborated this finding and proposed explanatory mechanisms [46–49], while none have shown a finding of sufficient significance to discourage use of the anesthetic.

On the other hand, the opioid analgesic tramadol could inhibit proliferation, migration, and invasion of breast cancers by inactivating the α2-adrenoceptor signaling pathway [50]. Based on cellular experiments, Huang et al. concluded that tramadol inhibits the progression of breast cancer cells and should be investigated further for use in combination therapy, especially for triple-negative breast cancer [51]. Like tramadol, the α2-adrenoceptor antagonist rauwolscine also suppressed tumor growth, in mice with human breast cancer cells [52]. Found in multiple botanical sources and marketed as a fat-burning nutritional supplement, it has also been shown to function as a 5-HT_1_ receptor partial agonist and 5-HT_2_ receptor antagonist.

### Acetylcholine

Acetylcholine (ACh) functions in the nervous system as a neurotransmitter at the autonomic ganglia, the parasympathetic innervated organs, and the neuromuscular junction between motor nerves and skeletal muscle. Acetylcholine receptors (AChRs) fall into one of two categories; the relatively slow activating G protein-coupled metabotropic muscarinic receptors (mAChRs) and the faster activating ionotropic nicotinic receptors (nAChRs). ACh helps regulate cellular proliferation, differentiation, and apoptosis.

Providing a partial explanation for the role of smoking in breast cancer, Nishioka et al. showed a decade ago with MCF10A (benign) and MDA-MB-231 (malignant) breast cells that when nicotine is ligated with nAChR, it promotes EGFR and Src signals [53]. Huang et al. showed in a murine model that advanced stage triple negative breast tumors are associated with higher levels of α9-nAChR gene expression [54]. α7-nAChR also appears to be up and it has been suggested that subtype-specific AChR antagonists could present an attractive pharmaceutical direction to prevent breast cancer progression [55].

Like nAChRs, the mAChRs also appear to influence breast cancer, specifically in that they are upregulated in breast tumors and absent in normal breast cells and tissues. Sales et al. has reported that mAChR agonists can act against breast tumors in a dose-dependent manner and be effective even at low doses [56].

### Serotonin

Serotonin (5-hydroxytryptamine, 5-HT) is synthesized from the essential amino acid tryptophan and mediates motility in the gastrointestinal tract and is a vasoactive agent in the blood. As a monoamine neurotransmitter, it also acts in the central nervous system. It regulates epithelial homeostasis in the breast. Serotonin is believed to impact immune signaling and stimulate growth of breast cancer cells [57–61]. Olfati et al. showed that in samples from breast cancer patients, 5HTR2A and 5HTR3A genes are more expressed in tumoral tissues than marginal tissues [62].

Serotonin also initiates angiogenesis by the proliferation, invasion, and migration of endothelial cells [63]. Sonier et al. found that serotonin promotes the growth and division of breast cancer cells, specifically MCF-7 cells, in part through the 5-HT_2A_ receptor. Proliferation and invasion is also facilitated by the 5-HT_7_ receptor in MDA-MB-231 cells. In these cells, 5-HT is essential to enhance the expression of TPH1 (tryptophan hydroxylase 1) and VEGF, supporting the mitogenic and oncogenic impact of 5-HT on breast cancer.

Tramadol, mentioned earlier in the context of α2-adrenoceptor, also interacts with serotonin receptors. Kim et al. reported that patients who received tramadol after breast cancer surgery had a decreased risk of postoperative recurrence and mortality, with the anti-tumor effect of tramadol appearing to involve inhibition of proliferation, induction of apoptosis, and effects on the serotonin 2B receptor and transient receptor potential vanilloid-1 expression [63]. More generally, antagonists of serotonin biosynthesis, transport, and activity appear to diminish breast cancer stem cell viability [64]. Serotonin production in a tumor sample may be a predictor of poor prognosis [65].

If lowering serotonin activity reduces breast cancer recurrence, then a logical question is the impact of medications that raise serotonin levels. Evidence has been conflicting on this long-standing question. Two decades ago, researchers reported that use of tricyclic medications was associated with significantly increased breast cancer risk [66, 67] and that use of selective serotonin reuptake inhibitor (SSRI) drugs may also pose a breast cancer risk [68, 69]. The SSRI fluoxetine may increase the number of breast cancer brain metastases at least in part due to inflammatory changes in the brain [70]. Supporting these contentions, this year researchers in Israel reported based on an analysis of 7000 patients that use of SSRIs in the years prior to breast cancer diagnosis, or in the years following diagnosis, was associated with substantially increased mortality [71].

However these studies were far from definitive and other analyses have reached the opposite conclusion. For example, a 2012 meta-analysis by Eom et al. on the relationship between SSRI use and breast cancer risk observed only marginal association attenuated over time, with no clinical significance [72]. Similarly, a recent meta-analysis by Li et al. reviewed 19 studies, finding no causal relationship [73]. And breast cancer cell line research has shown no significant effect of SSRIs on cell glucose uptake [74]. Indeed, other work has even suggested that such medications may even be used to positive effect. Murine research has indicated that inhibition of serotonin reuptake can inhibit breast tumor formation and that an SSRI given with docetaxel shrinks breast tumors [75, 76].

While the effectiveness of SSRIs for their primary indication may be in debate, there is little argument that they increase serotonin levels [77]. Further research on their impact on breast cancer is warranted, both to determine the direction of clinical impact of SSRIs in humans and to pursue serotonin receptor agonists as potential therapeutics [78].

### Dopamine

Breast cancer overexpresses the hormone prolactin, and prolactin is implicated in breast cancer growth, metastasis, and chemoresistance [79–81]. The hormone is under the inhibitory control of the neurotransmitter dopamine, raising the question of whether dopaminergic drugs can improve breast cancer outcomes. Dopamine is a catecholamine, like epinephrine and norepinephrine, and a precursor to their synthesis. It is important for regulation of behavior, movement control, endocrine, and cardiovascular function.

Dopamine or its receptor agonists seem to exhibit inhibitory effect on tumor growth in breast and several other cancer types. However, dopamine fails to diminish the proliferation and invasion of breast and colon cancer cells, indicating that factors such as tumor type, receptors expressed, and doses used play a role [82]. An important mechanism in dopamine’s tumor-suppressive effect is decreased angiogenesis. Activation of the DRD1/cGMP/PKG pathway induces growth arrest in vitro and causes tumor shrinkage and reduced bone metastasis in breast cancer [83].

A small study of giving patients with metastatic breast cancer cabergoline, an agonist of D2 dopamine receptors that has an inhibitory effect on pituitary prolactin secretion, had an inconclusive outcome [84]. Later, Goyette et al. found that phenothiazines, anti-psychotic drugs, reduced invasion and proliferation and increased apoptosis of triple negative cancer cells in vitro, by reducing PI3K/AKT/mTOR and ERK signaling [85]. Furthermore, they observed that administering phenothiazines to mice with triple negative breast cancer xenografts reduced tumor growth and metastasis. Other medications are being actively researched, with side effects being a significant consideration [86].

### Histamine

Best known for its role in allergies, histamine is an important monoamine neurotransmitter. Four G-protein-coupled histamine receptor subtypes mediate neuronal histamine’s effects [87]. The histamine H4 receptor, which is primarily expressed in immune cells and has also been found in breast cancer tissue, has been implicated in breast cancer growth [88–90]. Histamine decarboxylase expression level is correlated with relapse free and overall survival and histamine administered to mice with 4T1 triple negative tumor cells reduced tumor growth and increased apoptosis [91].

Speisky et al. reported that the H4 receptor may be a useful biomarker for predicting triple negative breast cancer prognosis [92]. Based on data from the Cancer Genome Atlas, they observed that the H4 receptor is downregulated in basal-like/triple negative breast cancer compared with luminal A and normal breast-like tumors. Furthermore, among basal-like/triple negative breast cancer patients, higher expression of the H4 receptor was associated with improved progression-free and overall survival outcomes. Further analysis of 30 triple negative breast cancer tumor samples showed that high H4 receptor expression in peritumoral tissue correlated with lesser lymph node involvement, unifocal triple negative breast cancer, and increased patient survival.

### GABA

The primary inhibitory neurotransmitter in the human brain is GABA. GABA may promote cancer cell proliferation and migration and is amplified in multiple cancers including breast. Gumireddy et al. observed that the GABA_A_ receptor alpha3, which is normally exclusively expressed in adult brain, is expressed in breast cancer, with higher tumor expression being associated with poorer survival [93]. As shown in mouse models, the receptor activates the AKT pathway, increasing breast cancer cell invasion and metastasis. Both Neman et al. and Dahn et al. have found that activation of GABA_A_ receptors increase brain metastases in breast cancer patients. The receptor could therefore represent a promising therapeutic target [94, 95].

Propofol is a drug with agonist activity for the GABA_A_ receptor and causes actin reformation and migration of breast cancer cells by collagen matrices. Observational epidemiological studies also show that benzodiazepine use increased the risk of breast cancer and many other cancers in a dose-dependent manner. Mimics of GABA, an inhibitory neurotransmitter, are frequently used to reduce peripheral nerve pain caused by chemotherapy. However, there is concern that GABA treatment could increase breast cancer metastasis [95]. Improved understanding will help ascertain if there are any adverse impacts from existing medication use.

### Neurotensin and neuropeptide Y

From 1419 primary breast tumors in a French institute, neurotensin receptor-1 overexpression was found in about one-third of breast tumors from patients undergoing primary surgery [96]. Neurotensin, activating neurotensin receptor, increases tumor cell proliferation, invasion, migration, and antiapoptotic effects [97]. Neurotensin receptor antagonists appear to ameliorate the situation and could provide a method for treating tumors overexpressing neurotensin receptors. Neurotensin is upregulated by estrogen in normal epithelial breast cells. In breast cancer cells, upregulation of the NTS-1 receptor leads to increased cellular migration and invasion. High expression of NTS-1 receptor has been associated with tumor grade, size, and number of metastatic lymph nodes [98].

Neuropeptide Y is a 36 amino-acid neuropeptide that plays a role in various physiological and homeostatic processes in the nervous systems including the osteogenic response. Its major receptors are overexpressed in multiple tumors including breast cancer metastasis [99]. Neuropeptide Y helps breast cancer cells proliferate and metastasize in part due to its role in angiogenesis via its effects on vascular smooth muscle and VEGF [100]. Li et al. elaborated on prior links between breast cancer and osteoporosis noting that Neuropeptide Y and its receptors are also involved in the regulation of bone metabolism [101]. They propose the Y1 receptor as a potential target for stem cell therapy to treat breast cancer and osteoporosis.

### MAOI synergy with PD-1/PD-L1 blockade therapy

Immune checkpoint blockade therapy has become a key tool in the treatment of several types of cancer, including triple negative breast cancer, in recent years. There is an active line of research towards improving and broadening the efficacy of such immunotherapies by modifying the tumor microenvironment. Neurotransmitters appear to play an important role here. Targeting tumor-associated macrophages to reduce their inhibition of antitumor T-cell reactivity may improve an immunotherapy’s activity against breast cancer [102].

Monoamine oxidase A is an enzyme bound to the outer mitochondrial membrane. In the brain, it is involved in degrading serotonin, dopamine, epinephrine, and norepinephrine [103]. Given its role in regulating the availability of serotonin and dopamine, there are small molecule monoamine oxidase A inhibitors (MAOIs) that are approved by the FDA for treatment of depression and Parkinson’s disease. Wu et al. demonstrated that MAOA induces the epithelial-to-mesenchymal transition (EMT) and stabilizes the transcription factor HIF1α, which promotes invasiveness and metastasis in prostate cancer [103]. High MAOA expression correlated with worse clinical outcomes. MAO A inhibitors reduced proliferation, microvessel density and invasion, and increased macrophage infiltration in drug-resistant tumors [104]. LaPierre et al. showed that MAO knockout mice had elevated markers of immune stimulation and decreased expression of markers of immune suppression compared MAO A wildtype [105]. They suggested that the deletion of MAO A reduces immune suppression in tumors to enhance antitumor immunity. Thus, MAO A inhibitors may alleviate immune suppression, increase the antitumor immune response and be used for cancer immunotherapy.

An excellent demonstration of this direction was provided by Wang et al. [106]. They reported last year on their investigation of the potential of monoamine oxidase A for reprogramming tumor-associated macrophages. Wang et al. found that monoamine oxidase A promotes tumor-associated macrophage immunosuppressive polarization and subsequent inhibition of antitumor immunity in mice. They showed that MAOI treatment suppressed tumor progression in preclinical mouse syngeneic and human xenograft tumor models, and furthermore that combining MAOI and anti-PD-1 treatments resulted in synergistic tumor suppression. The authors also conducted clinical data correlation studies and found that intratumoral monoamine oxidase A expression level was negatively correlated with patient survival in several cancers, including breast cancer in the GSE9893 cohort [106]. The combination of observations is suggestive that off label use of MAOIs could potentially suppress tumor metastasis and increase antitumor immunity.

## Discussion

Piecing together the puzzle of neurotransmitters’ influence on breast cancer is vital to better understand breast cancer biology and discover novel treatment approaches. It is also urgent to clarify the effects—positive, negative, or neutral —on breast cancer of commonly used medications, including beta blockers, SSRIs, MAOIs, and many more. Even the anesthesia used for breast cancer surgery may need thoughtful examination [107].

While all this potentially complicates the picture of breast cancer prevention and treatment, one reason that neurotransmitters provide a compelling framework for new approaches to breast cancer is the manner in which they link seemingly disparate observations and research directions. This is a source of intricacy, even outside the domain of pharmaceuticals. For example, exercise is advised as preventive against breast cancer, and cancers in general but is also a stressor that releases adrenaline and, as discussed, β2-adrenergic receptor signaling appears to be detrimental in regards to breast cancer. Jensen et al. propose combining exercise and beta blockers for breast cancer patients, such that the positive adrenergic signaling advantages of exercise can be obtained while avoiding chronic adrenergic signaling in the tumor microenvironment [108]. Wackerhage et al. suggest explanations for the seeming paradox of catecholamines’ varying effects [109].

Psychological stress has been observed to be a negative prognostic factor for survival among breast cancer patients [110] and beta adrenergic signaling provides a molecular mechanism to explain this [2, 111]. Addressing this factor could provide benefits at low cost. For example, a meta-analysis of studies of the effect of meditation on the psychological stress level of breast cancer patients found significant benefits in self-reported stress and molecular markers [112] and a randomized trial teaching techniques from cognitive behavioral therapy or relaxation training to women being treated for breast cancer showed reductions in stress and serum inflammatory markers [113].

Neurotransmitters may also provide an underpinning for observations related to the influence of the intestinal microbiome on breast cancer. The gut microbiota has been found to influence treatment side effects and prognosis in breast cancer patients [114]. This is consistent with the finding that intestinal microbiota impact regulation of various neurotransmitters in the body [115]. Indeed, dopamine, GABA, and the vast majority of serotonin in the body are all produced in the gut.

As indicated in prior sections and Table 4, there have been numerous clinical trials that relate to the role of neurotransmitters and breast cancer. These are essential, but further progress in our understanding would also be sped up by large-scale epidemiological studies. Prospective studies have their role, but given the commonality of factors such as the various medications discussed in this review, there is also a significant opportunity for further retrospective studies, both exploratory and to examine specific hypotheses. Access to larger patient corpora than are normally obtained should be sought to provide greater statistical significance.

**Table 4:** Selected clinical trials examining neurotransmitters effects on breast cancer from clinicaltrials.gov.

Trial title	Clinicaltrials.gov identifier	Neurotransmitter	Intervention	Recruitment status
Venlafaxine for hot flashes after breast cancer	NCT00198250	Serotonin and norepinephrine	Venlafaxine – SNRI, reduction in hot flashes	Completed − 2005
Solifenacin compared to clonidine for reducing hot flashes	NCT01530373	Acetylcholine	Solifenacin – muscarinic AChR antagonist, efficacy in reducing hot flashes	Active, not recruiting
Imipramine on ER + ve and triple negative breast cancer	NCT03122444	Serotonin and norepinephrine	Imipramine – TCA, changes in biomarkers and proliferation rate	Active, not recruiting
Individualized versus Standard Care for breast cancer patients at high risk for chemo-induced nausea and vomiting – ILIAD study	NCT02861859	Dopamine	Olanzapine – atypical antipsychotic, reduction in nausea	Completed − 2019
Study to assess effect of 8 wks of duloxetine therapy on breast cancer patients with aromatase inhibitor-associated pain	NCT01028352	Serotonin and norepinephrine	Duloxetine – SNRI, decrease in musculoskeletal pain and menopausal symptoms	Completed − 2011
Neoadjuvant propranolol in breast cancer	NCT02596867	Epinephrine and norepinephrine	Propranolol – non-selective Beta-blocker, change in proliferative index	Terminated − 2017
Propranolol hydrochloride in treating patients with recurrent or metastatic solid tumors that cannot be removed by surgery	NCT02013492	Epinephrine and norepinephrine	Propranolol – non-selective Beta-blocker, effect on growth of tumor cells (slowing by blocking the use of hormones), effects on tumor microenvironment and host immune system	Active, not recruiting
Impact of dexmedetomidine on breast cancer recurrence after surgery	NCT03109990	Epinephrine and norepinephrine	Dexmedetomidine – alpha2 adrenergic agonist, Increase in breast cancer recurrence and metastasis, effects on the immune system	Recruiting
Effects of dexmedetomidine on breast cancer cell function in vitro	NCT03108937	Epinephrine and norepinephrine	Dexmedetomidine – alpha2 adrenergic agonist, Effects on proliferation, migration, and metastasis in vitro	Completed − 2017
The effect of intraoperative dexmedetomidine on postoperative morphine requirements after breast surgery	NCT04454515	Epinephrine and norepinephrine	Dexmedetomidine – alpha2 adrenergic agonist, Effects on postoperative narcotics requirements	Recruiting
Cabergoline in metastatic breast cancer	NCT01730729	Dopamine	Cabergoline – dopamine agonist, Efficacy in treating metastatic breast cancer by lowering prolactin levels	Completed − 2017
Nicotinic treatment of post-chemotherapy subjective cognitive impairment	NCT02312934	Dopamine, serotonin, norepinephrine, acetylcholine, gamma-aminobutyric acid	Nicotine – nAChR agonist, Effect of transdermal nicotine on chemotherapy-related cognitive impairment	Completed − 2018

At the other end of the spectrum, insights can also be expected from advances in sophisticated molecular experimental techniques. McCallum et al.’s recording of neural activity in a triple-negative mammary cancer mouse model while the tumor grew and metastasized is a recent example [116]. Another example is the finding that in mice triple negative breast cancer tumors have more sensory neurons and the axon guidance molecule Plexin B3 mediates cancer cells’ adhesion and migration on sensory nerves [117].

Lastly, it is worth considering why neurotransmitters would have a substantial impact on breast cancer and whether it is reasonable for this to be the case. Indeed, a reason why this relationship was not meaningfully considered for decades is that it was not self-evident that there should be one. However, findings over recent years reveal the ubiquity of neurotransmitters in the body’s various signaling pathways. Evidence even suggests that certain neurotransmitter precursors evolutionarily predate the appearance of neurons and animals [118]. Neurotransmitters have come to play a number of roles as messengers, and there is little surprise that these signaling molecules would also be critical in the growth and spread of cancer.

Neurotransmitters appear to complicate the clinical and mechanistic picture of breast cancer, but denying this complexity is unlikely to pay dividends. Taking a signaling oriented view of breast cancer, particularly incorporating neurotransmitters, unifies various observations and provides clear direction for obtaining clinically significant short-term and long-term results in improving therapeutic strategies.
